# A Smart-Anomaly-Detection System for Industrial Machines Based on Feature Autoencoder and Deep Learning

**DOI:** 10.3390/mi14010154

**Published:** 2023-01-07

**Authors:** Imran Ahmed, Misbah Ahmad, Abdellah Chehri, Gwanggil Jeon

**Affiliations:** 1 School of Computing and Information Science, Anglia Ruskin University, Cambridge CB1 1PT, UK; 2 Department of Animal and Agriculture, Hartpury University, Gloucester GL19 3BE, UK; 3 Faculty of Health and Applied Sciences, University of West of England, Bristol BS16 1QY, UK; 4 Department of Mathematics and Computer Science, Royal Military College of Canada, Station Forces, Kingston, ON K7K 7B4, Canada; 5 Department of Embedded Systems Engineering, Incheon National University, 19 Academy-ro, Yeonsu-gu, Incheon 22012, Republic of Korea

**Keywords:** artificial intelligence, deep learning, industrial machine, anomaly detection

## Abstract

Machine-health-surveillance systems are gaining popularity in industrial manufacturing systems due to the widespread availability of low-cost devices, sensors, and internet connectivity. In this regard, artificial intelligence provides valuable assistance in the form of deep learning methods to analyze and process big machine data. In diverse industrial applications, gears are considered a condemning element; many contributing failures occur due to an unexpected breakdown of the gears. In recent research, anomaly-detection and fault-diagnosis systems have been the gears’ most contributing content. Thus, in work, we presented a smart deep learning-based system to detect anomalies in an industrial machine. Our system used vibrational analysis methods as a deciding tool for different machinery-maintenance decisions. We will first perform a data analysis of the gearbox data set to analyze the data’s insights. By calculating and examining the machine’s vibration, we aim to determine the nature and severity of the defect in the machine and hence detect the anomaly. A gearbox’s vibration signal holds the fault’s signature in the gears, and earlier fault detection of the gearbox is achievable by examining the vibration signal using a deep learning technique. Therefore, we aim to propose a 6-layer autoencoder-based deep learning framework for anomaly detection and fault analysis using a publically available data set of wind-turbine components. The gearbox fault-diagnosis data set is utilized for experimentation, including collecting vibration attributes recorded using SpectraQuest’s gearbox fault-diagnostics simulator. Through comprehensive experiments, we have seen that the framework gains good results compared to others, with an overall accuracy of 91%.

## 1. Introduction

Advancements in modern technology aim to enhance today’s industrial applications significantly; machine systems are now evolving to become more complex and complete critical tasks. Thus, it is necessary to monitor these systems’ conditions in order to improve their reliability. Any rotating machine, including pumps, compressors, steam turbines, and wind turbines, will ultimately reach a threshold of poor health conditions. One useful approach for improving their cost-effective maintenance and reliability is to use condition-monitoring and health-surveillance systems. These systems will aim to detect unexpected failures, faults, and anomalies in the machines [1,2].

Monitoring and prognostics systems developed on data-driven approaches need a sufficient amount of data, which is collected using a network of various kinds of sensors that monitor systems performance [3]. The data set collected from different machines through various sensors and devices normally has high dimensionality and sometimes also possesses undesirable interference and noise. The high-dimensional data set has an immediate effect on the training time and on the performance precision of deep learning methods. In order to deal with such problems, researchers mainly focus on reducing the input data or signal dimensionality by collecting useful features that hold the health condition information of machines.

Various techniques have been developed to achieve this goal. Some researchers used machine learning and handcrafted feature-based methods [4,5] such as obtaining temporal features by calculating the root mean square or peak-to-peak distance approach [6]. However, the primary features of a good-condition monitoring system and anomaly detection enable one to accumulate valuable characteristics and use these characteristics to determine the deteriorating state of machines by investigating the variation from the healthy (normal) behavior. In addition, handcrafted feature engineering-based techniques need domain details and knowledge, along with “trial and error” approaches. Nevertheless, the advent of artificial intelligence [7] and the recent improvement of deep learning methods [8] have transformed the feature-extraction and dimensionality-reduction methods. It is anticipated that hidden units obtain features that will define the data by piling up the layer to assemble deep autoencoders and decrease the number of units in the hidden layers.

Inspired by the successful results of artificial intelligence-based methods, we presented an intelligent framework for detecting anomalies in an industrial data set in this work. We also utilized the data-analysis method to investigate insights into the data set [9] because the most valuable features are known instantly from the data; hence, we avoided using trial and error methods. We presented an autoencoder-based model focusing on the data set’s most salient features and enhancing the system’s results. Furthermore, the autoencoder model mainly learns to match standard data points and does not contain anomalies in the trained model. The proposed framework demonstrates its relevance in machine-condition monitoring and anomaly-detection systems by considering it in relation to a real-world gearbox fault diagnosis data set (https://data.openei.org/submissions/623, accessed on 20 November 2022). The benefits of the autoencoder model comprise the capability to operate without preprocessing, feature selection, or manual-feature engineering. It does not restrict itself to the preidentified anomalies. It has the potential to identify new anomalies. We also demonstrate the time and feature-domain features used to teach the model to improve a detection model’s performance. First, the vibration signals are preprocessed, and later the training extracts features and rebuilds the signal for absolute-anomaly detection. In summary, the main contribution of the work is presented as follows:

- To present a combined framework for anomaly detection and condition monitoring using an industrial data set.
- To perform time and frequency analysis in order to gain insight of the data set through data visualization.
- To develop a window-feature-based autoencoder model for anomaly detection and condition monitoring.
- To perform a comparative analysis of the developed model with state-of-the-art methods.

The work presented in the paper is arranged as follows: an overview of different methods used for anomaly detection is presented in Section 2. Then, Section 3 discusses the details of the developed anomaly-detection framework. The data set used to evaluate the framework is also presented in this section. We also discuss the time- and frequency-based data analysis in Section 3. Furthermore, experimental results, along with evaluation parameters, are also addressed in Section 4. Lastly, the presented work with some future guidelines is concluded and summarized in Section 5.

## 2. Literature Review

Researchers studied machine learning and deep learning methods for anomaly-detection and machine-condition monitoring systems. For example, the authors of [10] presented a time-series-based anomaly-detection system for rotatory machine fault/anomaly detection. In data analysis, features and their generations are meaningful ideas in order to perform anomaly detection. Moreover, the selected features are vital for calculating dissimilarities in data, therefore identifying anomalies. Conventionally, feature extraction from the time and frequency domain has been utilized for monitoring or computing the anomalies of the machine [11].

Numerous feature-extracted methods are handled using conditions that are sensitive to irregularities. Investigators have attempted to improve the implementation of machine- and anomaly-detection techniques [12,13] by executing multivariate examinations while utilizing different features jointly. Xia et al. [14] calculated 29 bearing features by utilizing signal-processing methods [15]. Others have tried to perform this by creating components for detailed failure patterns and completing multivariate investigations based on them. For example, the authors of [16] made five attributes, each liable to a distinct failure method. Nevertheless, such handcrafted extracted features are not generalizable and fail to deliver good machine health data, specifically in unfamiliar circumstances.

Ali et al. [17] attempted to classify different bearing classes utilizing a collection of time-frequency domain components and artificial neural networks (ANN). [18] substituted the traditional feature extraction step by using 1-D convolutional neural networks. The method is performed using raw motor signals. The assessment conducted on bearing fault identification showed their approach’s superiority over conventional feature extraction techniques. [19] used an autoencoder for feature extraction and utilized this information for training a supervised detection model for fault classification. [20] explored various one-class classifiers, including k-means and nearest neighbors, to classify faulty rotor bars present in an induction motor. They figured the k-nearest neighbor approach displayed good results among other tested techniques.

Deep learning architectures, specifical autoencoders, are successfully used to classify anomalies and faults into distinct classes [21]. A probabilistic method for classifying anomalies in the natural gas consumption industry is presented in [22]. Nevertheless, the prediction approach indicates the consumption ranks utilizing different independent attributes and does not include the temporal information. Liu et al. [23] offered a fault-diagnosis system based on STFT and deep learning using Rolling bearing sound signals. Autoencoders have also obtained a valuable pattern from multiple sensor data [24]. Jiang et al. [25] presented frequency-domain-feature-based methods employing auto-encoders and traditional classification algorithms such as support vector machines and random forest to complete the classification job. Wang et al. [26] presented a deep-learning-based system for fault-relevant feature extraction and fault classification.

Mao et al. [27] proposed a novel autoencoder for fault recognition. Hoang et al. [28] suggested a a convolutional neural network for fault detection using a vibration image of a rolling bearing. However, the authors mainly focused on overcoming overfitting due to training data because of the limited available dataset. The authors of [29] presented an improved feature-based method with local binary patterns for bearing-fault diagnosis, which requires more training.

Researchers in [30,31], presented a review of various deep learning methods [32,33] used for the diagnosis of faults and anomalies in different machinery systems. In the literature, various feature and deep learning-based methods are developed by researchers. However, as discussed above, methods encounter one or several limitations: the need for accurately labelled data sets, labour-intensive manual-feature extraction, or a failure to integrate temporal information. In contrast, the presented artificial-based anomaly-detection framework first utilized data analysis to give insight into the data set. Finally, the autoencoder-based deep learning model trained over the vibration signals autonomously monitors the health condition of machines and identifies anomaly detection.

## 3. Materials and Methods

This work introduced an automated artificial-based framework using deep learning to detect anomalies in industrial machine data. In the presented work, we first perform simple data analysis in order to gain insight into the data set. Next, we perform a time- and frequency-domain analysis of the vibrational data set and then propose a deep-learning-based autoencoder model for detection purposes. In the following subsections, the details of the data set, data analysis, and developed deep autoencoder model are provided in detail.

### 3.1. Gearbox Vibrational Data Set

**Figure 1:**
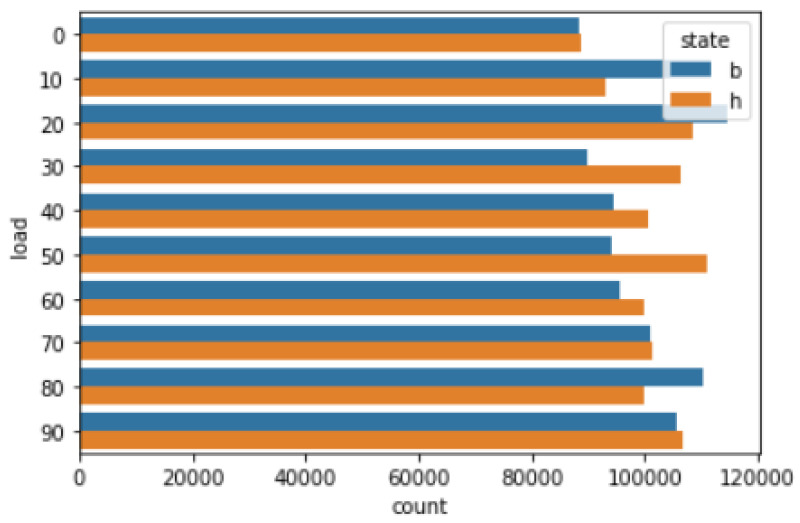
Total number of samples (healthy-condition *h* and broken-tooth condition *b*) in the data set.

The vibration signal data set utilized in this study is gathered from the (https://data.openei.org/submissions/623, accessed on 20 November 2022). It has been recorded with the help of four vibration sensors $a_{1},a_{2},a_{3},a_{4}$ placed in four different directions. The data set has been recorded under load variation from `0’ to `90’ percent. It includes the tooth data set in two scenarios: (1) healthy condition *h* and (2) broken tooth condition *b*. There are 20 files in total, 10 for the healthy gearbox and 10 for broken one. The data set is collocated by implementing SpectraQuest’s gearbox fault-diagnostics simulator. The gearbox device has configurations with various options and working behaviors. Based on these configurations, condition monitoring, gearbox working behavior, and vibration data is studied. The total number of samples for both healthy- and broken-tooth conditions for load 0 to 90 is shown in Figure 1. There are total four files for each sensor, and the files have roughly the same number of samples (85 k–120 k samples per file).

### 3.2. Time- and Frequency-Domain Analysis

**Figure 2:**
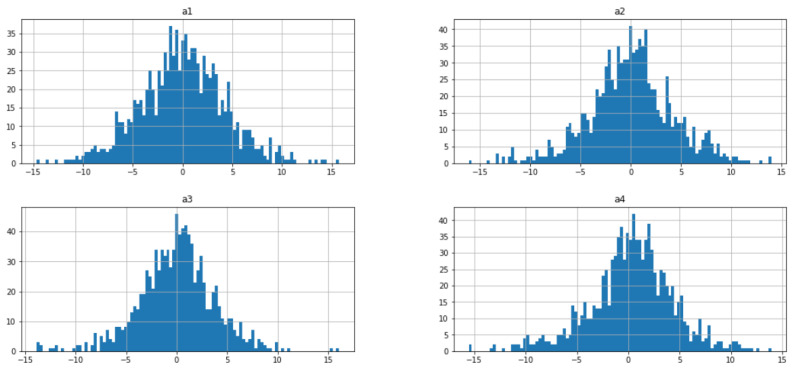
Histogram of all four sensors $a_{1},a_{2},a_{3},a_{4}$.

**Figure 3:**
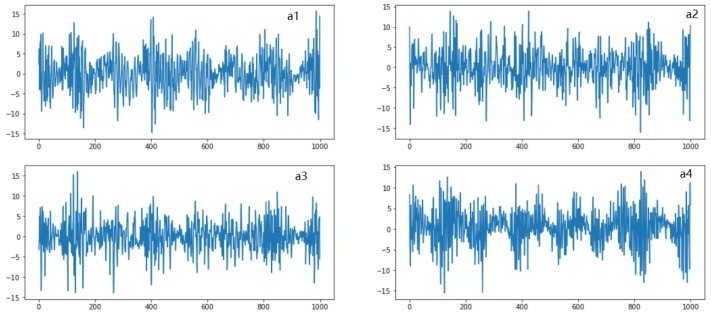
Time-domain analysis of each individual sensor e.g., $a_{1},a_{2},a_{3},a_{4}$.

In this section, we performed a time- and frequency-domain analysis of the data set in order to gain insight it. Through deep analysis, we understand the patterns of all four vibration sensors in both scenarios. First, in Figure 2, we have shown histograms of the four vibration signals. Each plot shows the histogram for each of the four sensor accelerations $a_{1},a_{2},a_{3},a_{4}$. It can be seen that all four sensors’ histogram amplitude values are different. In Figure 3, we have shown the time-domain analysis of all four sensors in both good- and broken-tooth scenarios. The upper row shows the plot for sensors $a_{1}$ and $a_{2}$, while the second row shows plots for sensors $a_{3}$ and $a_{4}$. It looks like there is a difference in amplitude for the sensor readings between the healthy gearbox and the one with the broken tooth. The difference between the amplitude values of all the sensors can be clearly seen against the time readings.

**Figure 4:**
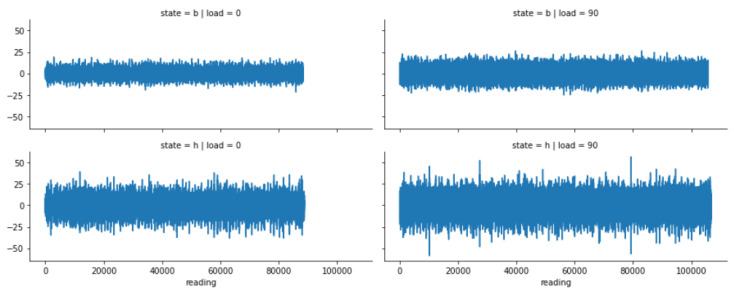
Time-domain analysis of different scenarios for load 0 and 90.

**Figure 5:**
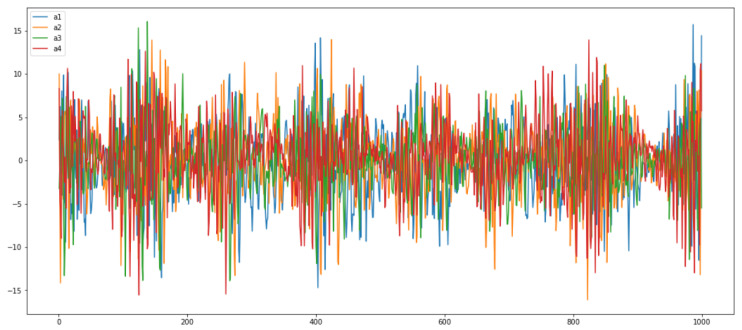
Time-domain analysis of all sensors $a_{1},a_{2},a_{3},a_{4}$.

In Figure 4, we have shown the time-domain analysis of two scenarios: the good- and broken-tooth scenario. It looks like there is a difference in amplitude between the sensor readings between the healthy gearbox and the one with the broken tooth. For load value 0, the machine shows some minor amplitude vibrations, which means that the machine is not working accurately, while for load 90, in a good or healthy state, the machine shows high amplitude vibrations, meaning that the machine is properly working without any anomaly. We also analyzed the combined plot of all four sensors in Figure 5 to show the readings’ distribution and compare the healthy and broken gearboxes for the same load and sensor. It can be seen that the values of all sensors are different.

**Figure 6:**
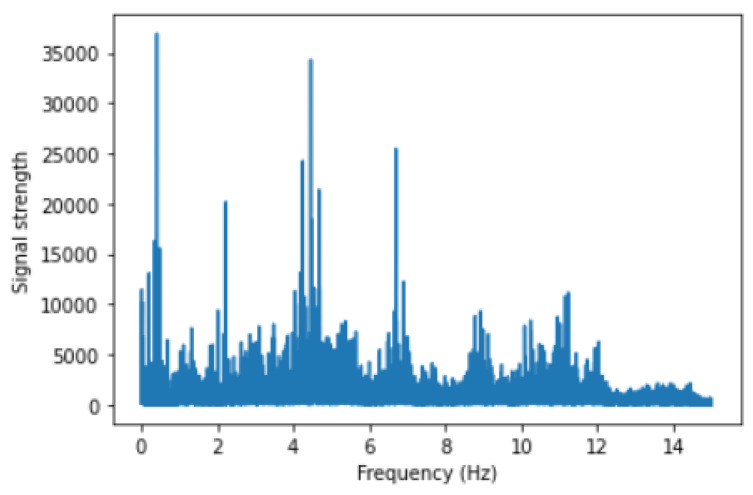
Frequency-domain analysis for all counted samples.

Looking at the vibration data across time is useful. However, as seen from the above figures, vibrations are occurring at different frequencies—this is very difficult to identify from the time-based analysis. Thus, the frequency analysis of the data may yield some more tangible features. In Figure 6, we have shown a frequency-domain analysis based on Fourier transforms to convert signal data (e.g., vibration readings) into its component frequencies. It can be seen that there is fluctuation at some points in the data (we can see some peaks).

**Figure 7:**
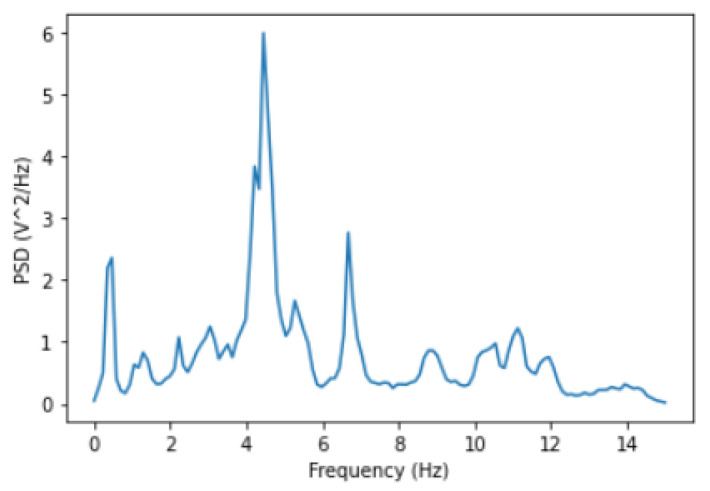
Power-spectral-density graph plotted using frequency values of the data samples.

**Figure 8:**
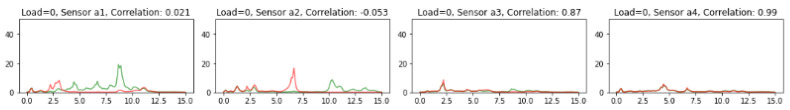
Power-spectral-density graph of all four sensors at load 0.

**Figure 9:**
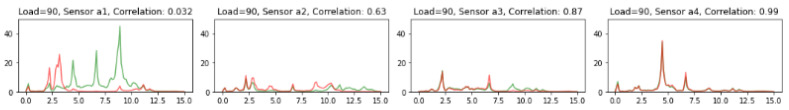
Power-spectral-density graph of all four sensors at load 90.

We plotted a power spectral density to clearly see these peak values as shown in Figure 7. At each load, compare the healthy and broken gearbox signals for each sensor. We can also correlate the power values for each frequency between the healthy and broken gearboxes to see how well they line up. The correlation between the two spectra is shown in the plot’s title for the sensors, and the signal power at the higher frequencies is much lower in the readings from the gearbox with the broken tooth. This pattern is visible across all loads. In Figure 8 and Figure 9, we have shown the power spectral analysis of all sensors using frequency values of the data samples for load ’0’ and ’90’. From the frequency spectrum the changes in the correlation values can be seen for load ’90’.

### 3.3. Developed Autoencoder Model for Anomaly Detection

**Figure 10:**
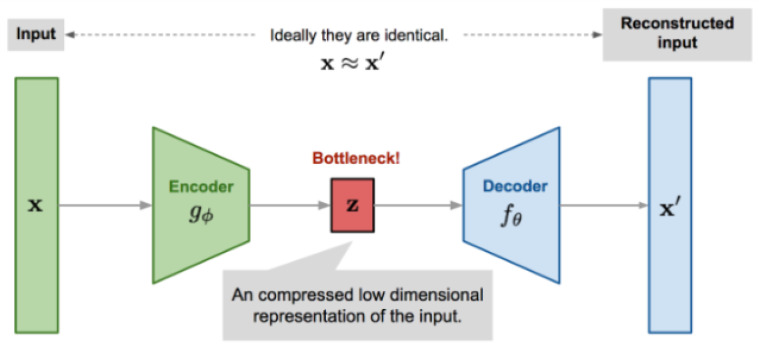
Illustration of autoencoder model architecture.

We proposed a 6-layer autoencoder-based deep learning model for anomaly detection in this work. The developed model comprises of encoder and a decoder steps. The encoder takes high-dimensional input data and translates it into low-dimensional data. On the other side, the decoder network receives the input from the encoder, that is, the output from the coder. The decoder’s goal is to rebuild the information that is provided as input data. The size of the output in this network is also larger than the size of the input. The overall architecture of the proposed model is illustrated in Figure 10. As discussed, that model receives high-dimensional information data and squeezes it down to the latent-space presentation in the bottleneck hidden layer. At the same time, the decoder accepts the latent presentation of the information as an input to rebuild the actual input data.

The encoder mainly encodes the input using the hidden layer to decrease the dimensionality of nonlinear and linear data; therefore, it is more powerful than principal component analysis. The model comprises two main functions: the encoder g(.) and a decoder f(.); each function is parameterized using ϕ and theta values, respectively. The low-dimensional value of the code is retained for input *x* through the bottleneck layers *z*, which is provided as

(1) $$z=g_{\phi (x)}$$

and the reconstructed input value is estimated as;

(2) $$x^{\prime }=f_{\theta }\left(g_{\phi (x)}\right)$$

In the above equations, θ, and ϕ are jointly learned to output, and a reconstructed data sample the same as the actual size of the input is obtained as;

(3) $$x=f_{\theta }\left(g_{\phi (x)}\right)$$

In other words, the equivalence function is learned. Different metrics are applied to quantify the discrepancy between two feature vectors, such as cross-entropy, which is applied when the activation function is sigmoid or a simple mean square root error (MSE) loss. The loss function for the autoencoder is estimated as follows:

(4) $$L_{\left(autoencoder\right)}\left(\theta ,\phi \right)=\frac{1}{n}\sum_{i}=1^{n}(x^{\left(i\right)}-f_{\theta }{\left(g_{\phi }\left(x^{\left(i\right)}\right)\right)}^{2}$$

**Figure 11:**
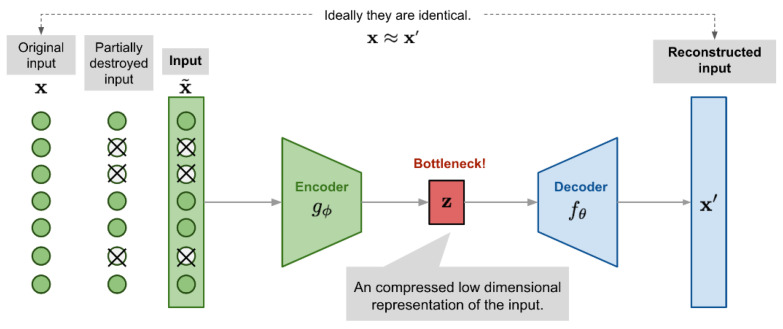
Illustration of denoising autoencoder model architecture.

Hence, the autoencoder learns through the equivalence function; we might face the threat of overfitting as there are more network parameters than the number of input data attributes. Thus, to overcome overfitting and enhance the robustness and performance of the model, we used the denoising autoencoder approach as shown in Figure 11. This approach is proposed as a modification to the autoencoder model. The input is partly corrupted by adding some noisy values or hiding some values of the input vector in a stochastic way;

(5) $$\hat{x}=M_{d}\left({\hat{x}}^{\left(i\right)}\;x^{\left(i\right)}\right)$$

In the above equation, $M_{D}$ defines the mapping from the actual data samples to the noisy ones. The final loss function is defined as follows:

(6) $$L_{\left(denoising_{-}autoencoder\right)}\left(\theta ,\phi \right)=\frac{1}{n}\sum_{i}=1^{n}(x^{\left(i\right)}-f_{\theta }{\left(g\phi \left(x^{\left(i\right)}\right)\right)}^{2}$$

In a high-dimension data set, anomalies are detected using the above discussed model. During the training, the normal input values are given to the encoder. The bottleneck layers use the latent representation of the actual input data. The decoder uses the output of the bottleneck layers to reconstruct the actual input data.

## 4. Results

**Figure 12:**
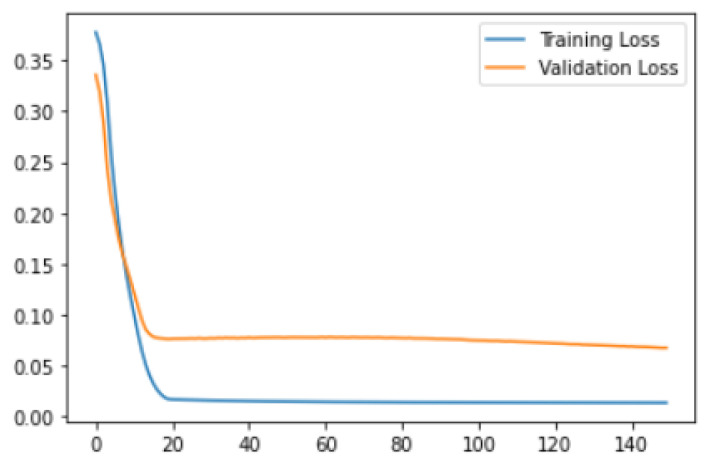
Training and validation loss.

The results of the above-discussed model are presented in this section. We trained the above model for 140 epochs. Firstly, we discussed training and validation loss observations as shown in Figure 12. In the figure, the x-axis shows the total number of epochs, while the y-axis shows loss values. It can be seen that the loss value decreases after the 20th epoch. The minimum value of the training loss is 0.01, while the validation loss is 0.07.

**Figure 13:**
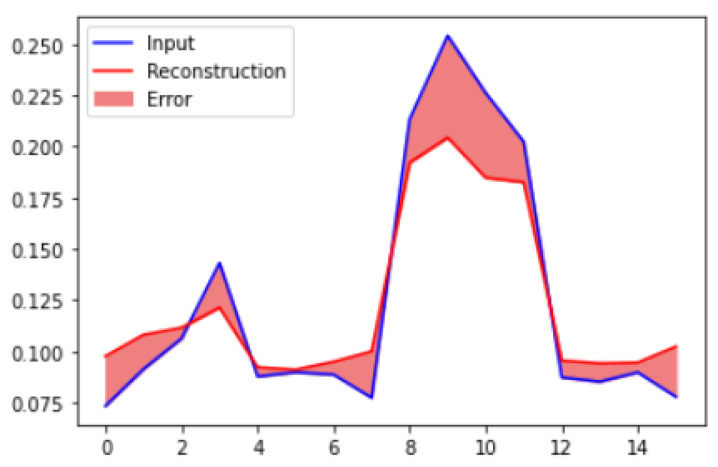
Reconstruction loss of normal data samples.

To estimate the reconstruction loss on the test data set, we anticipate the test data set values and compute the mean square error between the test data and the reconstructed test data. To calculate the reconstruction loss on test data, predict the test data and calculate the mean square error between the test data and the reconstructed test data. We plotted the reconstruction loss against the sample index. The results shown in Figure 13 are plotted for normal test data. It can be seen that the loss value is not so high for normal data set samples.

**Figure 14:**
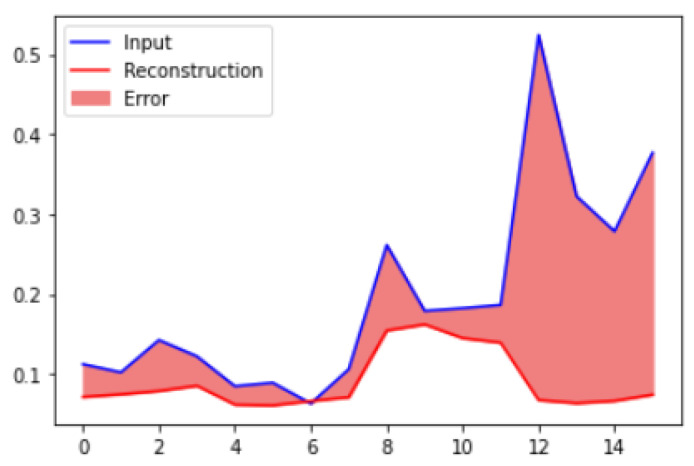
Reconstruction loss of anomalous data samples.

We also calculated the difference for the anomalous data set as shown in Figure 14. By comparing both figures, it can be seen that the difference in loss values between anomalous data sets is high. The peak value shows the detected anomalies as points highlighting the values where the reconstruction loss is high or greater than a defined fixed threshold value.

**Figure 15:**
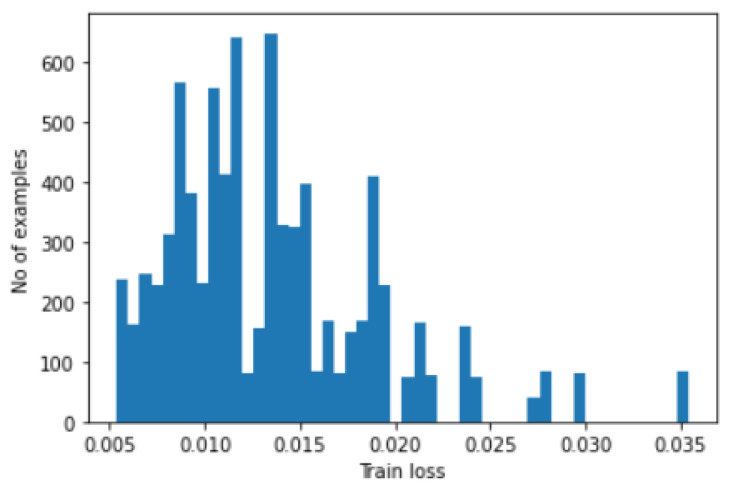
Histogram of train loss.

**Figure 16:**
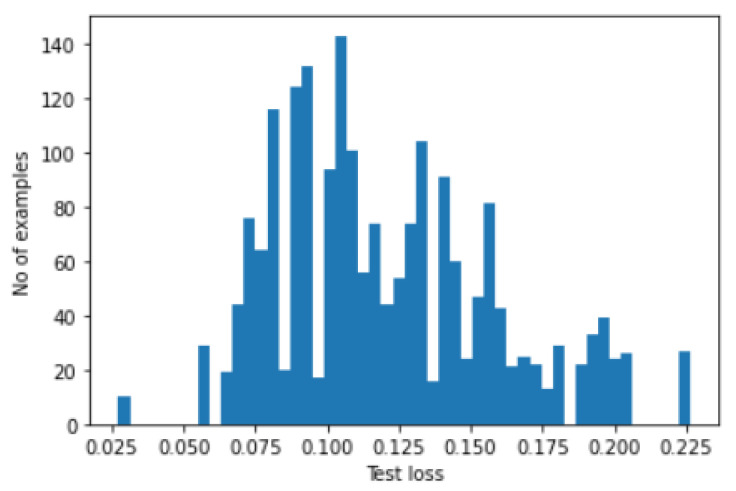
Histogram of test loss.

In order to find the threshold to fault detection, histograms are plotted for both train loss and test loss. In Figure 15, we have shown the plot of the train-loss histogram values, while in Figure 16, we have shown the plot of test loss histogram values.

The parameter evaluation results are discussed in Table 1. The model’s overall accuracy is 91%, the precision is 98%, and the recall value is 83%. We also compared results with other state-of-the-art methods. These methods are performed with manual-feature engineering and other machine learning methods. It can be seen that among all of the proposed methods, they display excellent results.

## 5. Conclusions

In this paper, we presented an artificial-intelligence-assisted deep learning method to analyze and process big machine data. We utilized vibrational analysis methods as a deciding tool for different machinery-maintenance decisions. We will first perform a data analysis of the gearbox data set to analyze the data’s insights. By calculating and examining the machine’s vibration, we determine the nature and severity of the defect in the machine and hence detect the anomaly. Our proposed model is based on a 6-layer autoencoder-based deep learning framework, which is used for the anomaly detection and fault analysis of wind-turbine components. The gearbox fault-diagnosis data set is utilized for experimentation; it includes the sets of vibration attributes recorded by SpectraQuest’s gearbox fault-diagnostics simulator. Through comprehensive experiments, we have seen that the framework achieves better results than other methods. The proposed method displayed excellent results, with an overall accuracy of 91%. In the future, we aim to extend this work to other deep learning models with different industrial benchmark data sets.

## Figures and Tables

**Table 1:** Comparison results with other state-of-the-art methods.

S.NO	Method	Accuracy	Precision	Recall
1	SVM	90%	94%	80%
2	Random forest	89%	91%	78%
3	KNN	84%	90%	76%
4	ANN	90%	94%	80%
5	Proposed autoencoder	91%	98%	83%

## Data Availability

Not applicable.
